# Microbial Community Structure and the Persistence of Cyanobacterial Populations in Salt Crusts of the Hyperarid Atacama Desert from Genome-Resolved Metagenomics

**DOI:** 10.3389/fmicb.2017.01435

**Published:** 2017-07-28

**Authors:** Kari M. Finstad, Alexander J. Probst, Brian C. Thomas, Gary L. Andersen, Cecilia Demergasso, Alex Echeverría, Ronald G. Amundson, Jillian F. Banfield

**Affiliations:** ^1^Department of Environmental Science, Policy, and Management, University of California, Berkeley, Berkeley CA, United States; ^2^Department of Earth and Planetary Sciences, University of California, Berkeley, Berkeley CA, United States; ^3^Ecology Department, Earth Sciences Division, Lawrence Berkeley National Laboratory, Berkeley CA, United States; ^4^Centro de Biotecnología, Universidad Católica del Norte Antofagasta, Chile

**Keywords:** metagenome, Atacama Desert, salt crust, hyperarid, hypersaline, environmental genomics, salar

## Abstract

Although once thought to be devoid of biology, recent studies have identified salt deposits as oases for life in the hyperarid Atacama Desert. To examine spatial patterns of microbial species and key nutrient sources, we genomically characterized 26 salt crusts from three sites along a fog gradient. The communities are dominated by a large variety of Halobacteriales and Bacteroidetes, plus a few algal and Cyanobacterial species. CRISPR locus analysis suggests the distribution of a single Cyanobacterial population among all sites. This is in stark contrast to the extremely high sample specificity of most other community members. Only present at the highest moisture site is a genomically characterized Thermoplasmatales archaeon (Marine Group II) and six Nanohaloarchaea, one of which is represented by a complete genome. Parcubacteria (OD1) and Saccharibacteria (TM7), not previously reported from hypersaline environments, were found at low abundances. We found no indication of a N_2_ fixation pathway in the communities, suggesting acquisition of bioavailable nitrogen from atmospherically derived nitrate. Samples cluster by site based on bacterial and archaeal abundance patterns and photosynthetic capacity decreases with increasing distance from the ocean. We conclude that moisture level, controlled by coastal fog intensity, is the strongest driver of community membership.

## Introduction

Microbial life has an amazing ability to survive extreme conditions. Environments once thought to be lifeless are now known to harbor organisms adapted to withstand a variety of physical and chemical challenges (Rothschild and Mancinelli, 2001). The hyperarid region of the Atacama Desert is one such example. Often described as the driest place on Earth, this region receives less than two mm of precipitation annually and was suggested by some to be the dry limit to life on Earth (McKay et al., 2003; Navarro-Gonzalez et al., 2003; Warren-Rhodes et al., 2006). More recently, microbial communities have been observed colonizing translucent salt crust on the surface of evaporitic deposits (Wierzchos et al., 2006). The key to microbial survival there is that halite deliquesces at an atmospheric relative humidity greater than 75%, creating a highly saline solution in its mineral pores (Wierzchos et al., 2012). Due to the frequency of marine fog intrusions in the hyperarid region, these salt crusts deliquesce on a regular basis (Cereceda et al., 2008; Finstad et al., 2016).

There is now considerable interest in the microbial community membership and structure of this ultra-dry environment. Early research identified cyanobacteria (*Chroococcidiopsis*) as a key autotrophic component to this system and demonstrated activation of photosynthetic systems during wetting events (Wierzchos et al., 2006, 2012; Davila et al., 2008, 2013). More recent studies using 16s rRNA sequencing have expanded our knowledge of abundant community members, profiling a variety of halophilic Bacteroidetes, Halobacteriales, and Proteobacteria (de los Rios et al., 2010; Stivaletta et al., 2012; Robinson et al., 2014). Higher resolution studies utilizing metagenomic techniques are required to more fully characterize community structure and to explore the metabolic strategies employed in this hyperarid and hypersaline environment (Crits-Christoph et al., 2016).

Here, we conduct the most in-depth sampling to date of the microbial communities in salt crusts of the Atacama Desert. Applying assembly-based metagenomic techniques to 26 samples at three sites along a fog gradient, we analyze the spatial patterns of community membership and genetic relatedness of species. Circumventing biases associated with amplicon-based sequencing, we detect low abundance phyla previously unreported in this system. Additionally, we examine evidence for strain dispersal and begin to characterize the metabolic capacities of members involved in key nutrient cycles.

## Materials and Methods

### Field Sites and Sample Collection

**FIGURE 1:**
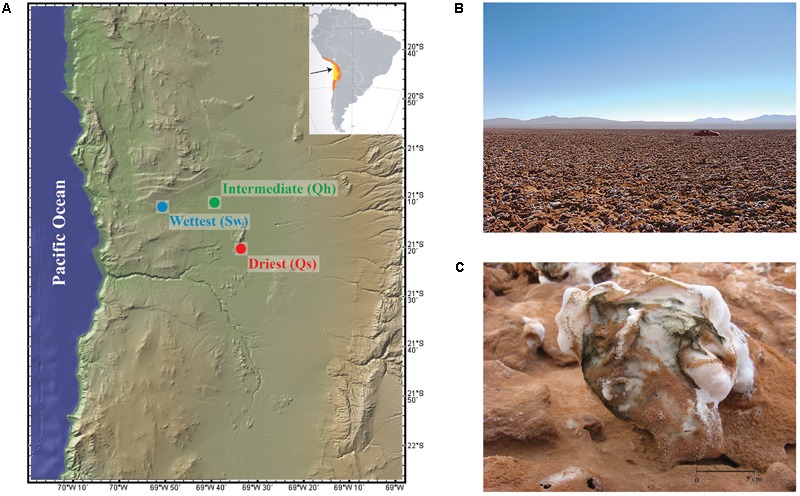
**(A)** Location of the field sites within the Atacama Desert. Yellow coloration on inset map indicates the location of the Atacama Desert and orange coloration indicates the extent of the arid region, **(B)** landscape view of the rugged salt crusts that cover the Salar Llamara, and **(C)** close-up view of the salt crusts with dark green pigment from the microbial communities. Map from GeoMap App (http://www.geomapapp.org/).

Within the Salar Llamara of northern Chile, three sites along a fog-delivered moisture gradient were selected for study (**Figure 1A**). Fog originates over the Pacific Ocean and moisture intensity and frequency therefore decrease with increasing distance from the coast. The wettest site is 25.5 km from the coast, the intermediate site is 44.1 km from the coast, and the driest site 54.2 km from the coast.

At all sites, a rugged and dynamic halite crust (NaCl) covers the surface. The salts have either originated from the evaporation of shallow groundwater from dry lakebeds (driest and intermediate sites) (Finstad et al., 2016) or are part of a regional Miocene halite deposit (wettest site) (Pueyo et al., 2001). Regardless of the salt source, the surface crusts are indistinguishable and similar processes involving interactions with wind and fog currently maintain them (**Figures 1B,C** and Supplementary Table 3).

Samples were collected in June 2013. At each site, three halite samples were selected from within three 3 × 3 m plots positioned 20–70 m apart from each other, totaling nine samples per site. Samples within the plots were chosen at random and removed using a sterilized rock hammer. Samples were stored in Whirl-Pak bags at room temperature for ∼14 days during transportation to the lab, at which point they were placed in a -20°C freezer until further processing. One sample from the intermediate site was used to optimize DNA extractions and therefore data from only eight of the nine collected samples from this site are reported on here.

A Decagon Devices Inc. leaf wetness sensor was installed at each site in June 2013 and collected continuous hourly data until August 2014. The leaf wetness sensors served as a proxy measurement for fog and dew accumulation. Because it did not rain during the year of study, we concluded that any “wetness” events recorded by the leaf wetness sensor were from dew or fog. A value of 460 counts from the sensor was chosen as the threshold to report wetness, which according to the manufacturer is a conservative value.

### DNA Extraction and Sequencing

Using sterile tools, samples were removed from their bag and placed on a sterilized surface. 20 g of sample was dissolved in nuclease-free water in sterile falcon tubes then filtered through an Amicon Ultra 30,000 NMWL centrifugal filter. A MoBio PowerSoil kit was used to extract the DNA from the filter.

The quality of DNA extracted was evaluated using a NanoDrop 2000 Spectrophotometer. All samples had a 260/280 ratio between 1.6 and 2.0. Samples were run in a 1% agarose gel with the DNA Molecular Weight Marker II (0.12–23.1 kbp) to screen for DNA degradation. The quantity of DNA extracted was measured using a Qubit 2.0. Library preparation and sequencing were performed at the UC Berkeley QB3 Sequencing Center. Wafergen PrepX DNA library preparation kits were used to ligate universal adapters and the Kapa enzyme and 13 cycles of PCR used to enrich for adapter-ligated fragments and to apply a differential index to the samples. Samples were run on an Illumina HiSeq2000 with 100 base paired end reads with a library size of 600–1000 base pairs.

### Assembly and Genome Reconstruction

Reads were trimmed and quality checked using SICKLE Version 1.21^1^ with default parameters, then assembled into scaffolds using IDBA_UD (Peng et al., 2012). Coverage of the scaffolds was determined using Bowtie2 with the –sensitive setting (Langmead et al., 2009). Open reading frames (ORFs) were predicted using the prodigal software (using metagenome settings, “-p meta”) (Hyatt et al., 2010). Genomes from metagenomes were binned using differential coverage information as described in Sharon et al. (2013). Briefly, coverage of each scaffold was determined by cross-mapping the reads of each sample against one assembly (Bowtie 2, –sensitive setting; Langmead et al., 2009). Genomes were binned from emergent self-organizing maps (ESOMs with a window size of 3 kb) and manually curated to remove scaffolds with anomalous GC content, coverage, or taxonomic classification (Wrighton et al., 2012). Genome completeness was estimated based on 51 bacterial and 38 archaeal single copy genes, as described previously (Probst et al., 2017). Predicted ORFs from prodigal were compared to KEGG (June, 2015), UniRef100 (July, 2014), and UniProt (June, 2015) sequence databases using Usearch (version 7.0.959^2^). All metagenome data from each sample was loaded into ggKbase^3^. ggKbase binning tools and genome summary visualizations were used to investigate contig binning and display metabolic information for each organism. Raw sequencing reads can be accessed through the National Center for Biotechnology (NCBI) BioProject PRJNA351262.

### De-Replication of Genomes at Species Level

Genomes with at least 70% completeness were de-replicated following Probst et al. (2017), and only the most complete genomes were used for further analysis. Briefly, for each genome bin, scaffolds were individually aligned (≥98% nucleotide identity) against each scaffold of another genome bin in the set. Genomes that shared over 70% of nucleotides were grouped together and the best representative genome was selected based on the number of archaeal/bacterial single copy genes, multiple single copy genes by using the following equation: score = (number of single copy genes)-2 × (number of multiple single copy genes). In case of a tie the genome with the greatest length was selected. Genomes can be accessed through the NCBI BioProject PRJNA351262.

### Hidden Markov Models Ribosomal Protein S3 Analysis

Ribosomal protein S3 subunits (rpS3) for all three domains (archaea, bacteria, and eukaryotes) were derived from the dataset used for a recent reconstruction of the tree of life (Hug et al., 2016). Proteins of the three domains were separately aligned using MUSCLE (Edgar, 2004), end-trimmed, then used to build hidden Markov models (HMM). Length cutoffs (min 120, max 450 amino acids) and score cutoffs (bacteria 111, archaea 172, and eukaryotes 175) were determined from the original dataset and refined against UniRef (UniProt Consortium, 2015) and in-house metagenomes containing archaea, bacteria and eukaryotes. Resulting rpS3 genes were clustered using USEARCH (cluster_fast; Edgar, 2010) and coverage of rpS3 genes was set to the coverage of the assembled scaffold (determined for each metagenome sample, see above).

### Statistical Analysis of Metagenomics Data

Relative abundances of microbial species were determined by stringently cross-mapping metagenomic reads onto the de-replicated set of genomes (allowing for only three mismatches, which is equivalent to 97% similarity to the template). Coverage per nucleotide of each genome was determined and then sum normalized across all samples using the total number of reads used for the mapping.

Multivariate analyses of the community data were performed as described in Weinmaier et al. (2015) using the R programming environment (R Core Team, 2015). In brief, community dissimilarities were calculated using the Bray–Curtis index and community structure was displayed using non-metric multidimensional scaling (NMDS). A multi-response permutation procedure (MRPP) of the datasets was done. For the metagenomic dataset, the chance corrected within-group agreement *A* = 0.2066, and significance of delta = 0.001. For the PhyloChip dataset the chance corrected within-group agreement *A* = 0.3462, and significance of delta = 0.005. Abiotic factors influencing the community were evaluated using a BioENV and PERMANOVA (Adonis). Factors included distance from the coast, hours of moisture (from the leaf wetness sensors), and the longitude and latitude of the sampling site. A Mantel test was performed (n repeats = 999) with matrices of sample distance based on GPS sampling coordinates and dissimilarities from the Bray–Curtis index.

### 16S rRNA Gene Analysis Using PhyloChip G3

16S rRNA gene amplification for PhyloChip analysis was performed as described in Hazen et al. (2010). The metagenomic template for the PCR reactions was 2 ng. Resulting amplicons were prepared and processed for PhyloChip analysis similarly to Brodie et al. (2006, 2007) and DeSantis et al. (2007). Two hundred and fifty ng of bacterial PCR product and 50 ng archaeal PCR product were applied to a G3 PhyloChip^TM^ (Second Genome, South San Francisco, CA, United States) following previously described procedures (Hazen et al., 2010). Only perfect matching fluorescence intensity from probes observed as responsive in at least four experiments were exported from all experiments then rank-normalized in Sinfonietta software and used as input to empirical probe set discovery as described in Probst et al. (2014). Relative abundances of OTUs were used for statistical analyses (NMDS) as described above for metagenomic data.

### Auto-Fluorescent Cell Count

Two or three samples from each site were collected and sectioned into three parts: upper, middle, and lower. A weighed piece of sample from each section was placed in a Falcon tube with a measured volume of water containing 1% TWEEN-20 and stirred for 1 h. One mL of the solution was then filtered using a Millipore polycarbonate filter and the filter mounted with a drop of mounting oil (Vecta-Shield) on a coverslip. The sample was observed in an Olympus FV1000 confocal microscope, Inverted IX81 microscope with 100 × /1.4 oil immersion objective at the Centro de Biotecnología at the Universidad Católica del Norte, Chile. Samples were illuminated with blue light (<450 nm) and/or green light (<540 nm) and total auto-fluorescent cells were counted in an area of 100 × 100 μm. An analysis of variance (ANOVA) was used to determine if differences in cell count between the upper, middle, and lower sections in a sample were significant. For those samples with a significant difference, a Tukey paired comparison test was used to determine which zones were significantly different.

### Detection of Nitrogen Fixation Pathways

We identified 14 HMMs in the TIGRFAM database^4^ and ran hmmsearch (HMMER version 3.1b2^5^) on all proteins from the assembled metagenomes using the built-in noise cut-offs for these HMMs.

### CRISPR Analysis

Analysis of CRISPR loci in Cyanobacteria was performed by searching the reconstructed genomes for CRISPR loci using CRISPRfinder online program^6^ (Grissa et al., 2007). The loci were classified into locus types based on shared Cas protein sequences, the repeat sequence, and shared spacers.

## Results

We reconstructed 115 distinct (de-replicated) draft quality genomes (>70% complete, 44 bacteria, and 71 archaea), of which 53 are high quality drafts (>90% complete, 18 bacteria, and 35 archaea) and one is closed and complete (Supplementary Table 1). Forty-three percent of the ∼150 × 10^9^ base pairs of sequence from all samples are accounted for by these genomes. In addition, we utilized ribosomal protein S3 (rpS3) to identify and taxonomically characterize the community composition. In total, we identified 198 unique rpS3 sequences, 155 archaeal, and 43 bacterial.

**FIGURE 2:**
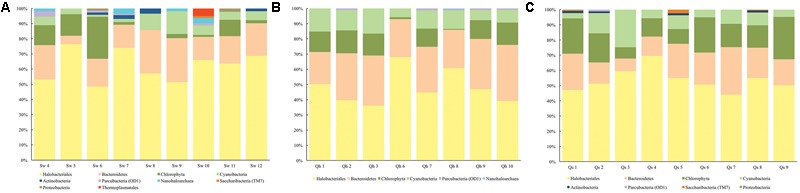
Composition of the community in each sample from ribosomal protein S3 data of the assembled metagenomic reads at the **(A)** wettest site (Sw), **(B)** intermediate site (Qh), and **(C)** driest site (Qs).

**FIGURE 3:**
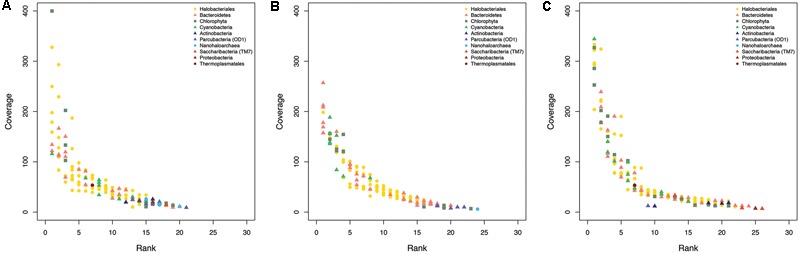
Rank abundance curves from ribosomal protein S3 coverage from the assembled metagenomic reads at the **(A)** wettest site, **(B)** intermediate site, **(C)** and driest site.

In all samples, communities are dominated by Halobacteriales, Bacteroidetes, algae (Chlorophyta), and Cyanobacteria (**Figures 2, 3**). Remarkably, based on analyses of the 198 rpS3 sequences, 72% of these organisms are found only in one sample and 84% are found only at one site (**Table 1**).

**Table 1:** Taxonomy of the **(A)** 115 reconstructed draft genomes (>70% complete) and the **(B)** 198 ribosomal protein S3 sequences identified from all 26 samples (9 at the wettest site, 8 at the intermediate site, and 9 at the driest site).

	Total	Site	Sample	Wet	Intermediate	Dry	All	Wet and	Intermediate	Dry and
Taxonomy	populations	specific (%)	specific (%)	only (%)	only (%)	only (%)	sites (%)	Intermediate (%)	and Dry (%)	Wet (%)
**(A)**
Halobacteriales	65	63	8	3	3	14	43	28	3	6
Bacteroidetes	31	45	–	13	23	10	45	–	6	3
Parcubacteria (OD1)	4	100	100	75	25	–	–	–	–	–
Actinobacteria	2	–	–	–	–	–	–	–	–	100
Nanohaloarchaea	5	100	100	80	20	–	–	–	–	–
Cyanobacteria	3	67	–	33	–	33	33	–	–	–
Thermoplasmatales	1	100	100	100	–	–	–	–	–	–
Proteobacteria	1	100	–	100	–	–	–	–	–	–
Saccharibacteria (TM7)	3	100	100	33	–	67	–	–	–	–
**(B)**
Halobacteriales	148	86	75	33	22	31	3	5	3	2
Bacteroidetes	23	65	43	30	26	9	17	4	9	4
Parcubacteria (OD1)	10	100	100	40	50	10	–	–	–	–
Actinobacteria	3	67	67	33	–	33	–	–	–	33
Nanohaloarchaea	6	100	100	100	–	–	–	–	–	–
Cyanobacteria	3	67	–	33	–	33	33	–	–	–
Thermoplasmatales	1	100	100	100	–	–	–	–	–	–
Proteobacteria	3	100	100	67	–	33	–	–	–	–
Saccharibacteria (TM7)	1	–	–	–	–	–	–	–	–	100

*Listed is the percent of populations from each taxonomic group found only at one site (site specific) or found only in a single sample (sample specific), and the percent of shared populations listed as either being at one site, all of the sites, or a combination of two sites.*

### Distribution of Cyanobacteria Based on CRISPR Loci

**FIGURE 4:**
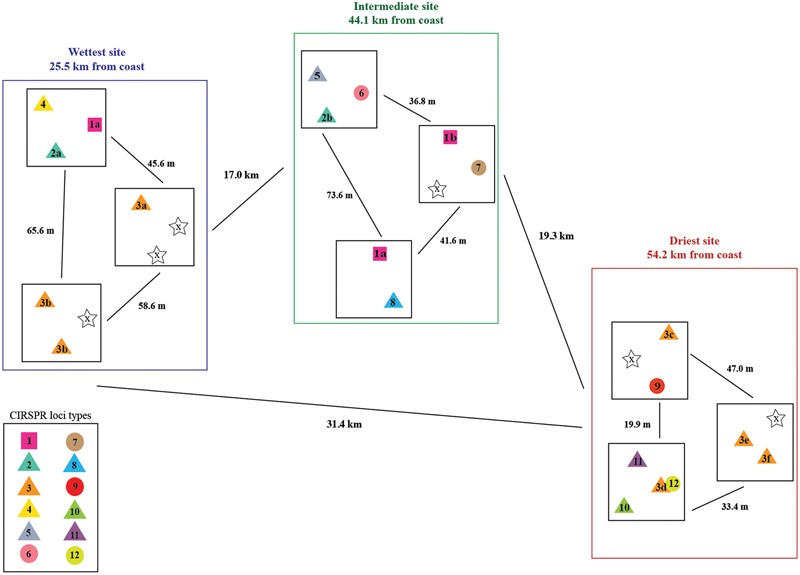
Distribution of the CRISPR loci types from the widely distributed Cyanobacteria. The 12 locus types are differentiated by symbol color and number (1–12). The shape of the symbol indicates which of the three repeat sequences was identified in that sample (circle, square, or triangle), and a star symbol indicates no CRISPR locus was found. Lower case letters are used to note variations within a single locus type due to either missing sequence or different terminal spacers (for example, 1a and 1b differ due to four additional terminal spacer sequences found in 1a but not in 1b). Distances between the sites and sampling plots are noted, but not drawn to scale.

We reconstructed three Cyanobacterial draft genomes, two Halothece and one Euhalothece. The Euhalothece is found in three samples at the wettest site and one of the Halothece found only in two samples at the driest site. In contrast to this limited range, the other Halothece is present in all communities at all sites (**Table 1**). To examine the genetic relatedness of this widely distributed Cyanobacteria, we compared the CRISPR locus of the 26 reconstructed genomes, one from each sample. We were able to identify CRISPR loci in 20 of the genomes, and classified them into 12 locus types based on shared Cas protein sequences, the repeat sequence (three in total), and shared spacers (**Figure 4**).

Notably, the CRISPR locus of one population at the wettest site is identical to that of a population at the intermediate site. Another sample at the intermediate site has a population that differs from these only due to the absence of three terminal spacer-repeat sets (locus group 1 in **Figure 4**). Locus type 2 includes another population that is similarly shared between the wettest and intermediate sites, and locus group 3 includes populations found at both the wettest and driest sites. In both of these locus groups, the terminal spacer inventories only slightly differ between populations at the different sites. The remaining nine locus groups are either distinct or share only one spacer.

### Distribution of Other Notable Organisms

The communities show an extremely high diversity of halophilic archaeal species, mostly Halobacteriales (**Table 1**). We reconstructed 65 draft genomes and identified 148 different rpS3 sequences from this group. Additionally, we reconstructed 31 Bacteroidetes draft genomes and identified 23 rpS3 sequences.

At lower abundance in the communities, we detected Nanohaloarchaea, OD1 (Parcubacteria), and TM7 (Saccharibacteria). Six distinct rpS3 sequences of Nanohaloarchaea were identified and five draft genomes were reconstructed. Remarkably, all of these Nanohaloarchaea genomes and rpS3 sequences are found to be sample specific (not shared between any two samples), and the vast majority are found only at the wettest site (**Table 1**).

To our knowledge, this is the first report of Candidate Phyla Radiation bacteria in hypersaline environments. We reconstructed four distinct draft Parcubacteria (OD1) genomes and identified ten unique rpS3 sequences. Similar to the Nanohaloarchaea, the majority were found at the wettest and intermediate sites. We reconstructed three Saccharibacteria (TM7) draft genomes and identified one unique rpS3 sequence. None of the Candidate Phyla Radiation bacteria were found to overlap between samples.

A near-complete genome of a novel Thermoplasmatales archaeon affiliated with Marine Group II was reconstructed from the wettest site. The rpS3 sequence belonging to this organism was identified and found in only one sample at the wettest site.

### Spatial Variation in Community Structure

**FIGURE 5:**
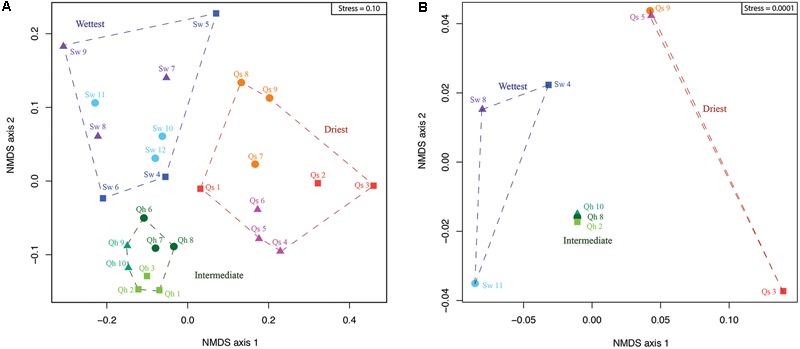
Community structure displayed using non-metric multidimensional scaling (NMDS) of the **(A)** 115 genomically defined organisms and **(B)** PhyloChip data. Samples taken from the same 3 m × 3 m plot are identified with the same color and symbol. Samples from the same site cluster together as do samples from the same plot at the intermediate and driest sites. Sw refers to samples from the wettest site, Qh samples from the intermediate site, and Qs samples from the driest site.

To compare the entire microbial community recovered from our metagenomic analysis of each sample, we used the abundance patterns of the 115 genomically defined organisms. Our results indicate that the microbial communities from samples at the same site are generally more similar than those from other sites (**Figure 5A**). In the ordination analyses, samples from the wettest and intermediate sites separate from those at the driest site along NMDS axis 1. Along NMDS axis 2, the wettest site forms a separate cluster from samples at the intermediate site.

Communities from samples collected at the intermediate site show the highest intra-group similarities relative to the other sites. Moreover, each of the three sampling plots at the intermediate site form individual clusters within this grouping. Samples from the wettest site show the highest within-site variation and low to no grouping of plots. Samples from the driest site show a trend for plot-based grouping on either NMDS axis 1 or 2.

Statistical analysis of the 16S rRNA gene amplicon data (PhyloChip) largely confirmed the results observed using the metagenomic data. A NMDS shows that samples group together by site and the intermediate site has the highest within-group similarity (**Figure 5B**). Along NMDS axis 1, it is apparent that samples from adjacent sites are more similar to each other than samples from opposing ends on the transect (e.g., the wettest site was closest to the intermediate site and furthest from the driest site).

A Mantel test of the distance between samples and community dissimilarity showed that the spatial distance between samples correlates with the dissimilarity of the microbial communities (*p*-value = 0.001 and observation value = 0.59, based on 999 replicates).

### Environmental Influence on Community Composition

A leaf wetness sensor was used as a proxy for the presence of moisture (dew or fog) at the sites. The results show that the wettest site received 3,113 h of fog, the intermediate site received 1,560 h of fog, and the driest site received 891 h of fog during the 9,504 h of monitoring.

We used statistical methods to link environmental conditions to the observed community composition patterns. Among the measured abiotic factors, moisture and longitude (distance from the Pacific Ocean) were determined to have the highest correlation with the observed microbial community patterns (BioENV correlation = 0.58). These two factors were also shown to have a significant influence on the observed dissimilarities of the samples (PERMANOVA, *p*-value < 0.001).

### Capacity for Carbon and Nitrogen Fixation

The key role of carbon fixation in all samples is assigned to Cyanobacteria and algae, likely with varying contributions across the communities and sites. To understand the capacity of the communities to sustain growth, a cell count of the photosynthetic organisms was performed. We found that the number of primary producers is greatest at wettest site and declines along the fog gradient. The average cell concentration per gram of sample was 1.53 × 10^7^ at the wettest site, 1.01 × 10^7^ at the intermediate site, and 3.03 × 10^6^ at the driest site. There were significant differences in cells between the upper, middle, and lower sections in all of the samples at the wettest and intermediate sites, and no significant differences between the sections in samples at the driest site (Supplementary Table 2). No nitrogenase-related genes were identified in any of our communities.

## Discussion

### Spatial Patterns of Species Diversity

The most striking finding of this study is the widespread distribution of a single very closely related Cyanobacteria (Halothece) in contrast to the extremely high sample specificity of most other community members. The presence of Cyanobacterial populations with identical CRIPSR loci at different sites strongly indicates recent dispersal of these organisms among the sites. Furthermore, the presence of loci that differ only at the terminal end likely indicates recent population divergence resulting from the acquisition of new spacers. The existence of 12 locus types overall suggests some population-level variety exists across this region of the Atacama, but this is minimal compared to that seen, for example, in the Halobacteriales and Bacteroidetes. Cyanobacteria are not commonly found in environments with salinities above 25%, and only the Aphanothece–Halothece–Euhalothece cluster are adapted to life at salt concentrations approaching NaCl saturation (Oren, 1994, 2008, 2015). This likely has limited the variety of Cyanobacteria able to colonize the salt crusts. Other studies of salt crusts in the Atacama Desert have also identified Cyanobacteria from this cluster (Wierzchos et al., 2006; de los Rios et al., 2010).

In contrast, the Halobacteriales order is comprised entirely of halophilic archaea, so it is not surprising that a wide diversity is present in the salt crust communities (Oren, 2008). However, the extremely high variety (an average of 43 different Halobacteriales rpS3 sequences per site), in combination with the high occurrence of site-specific sequences (75%), was unexpected. We suggest that organisms are introduced to the desert by atmospheric deposition or through evaporation of shallow groundwater, followed by selection for salt-adapted organisms. The high diversity of halophilic archaea may arise because the crusts are “banks” of introduced strains capable of at least one episode of growth, followed by preservation in the salt. We suggest a similar explanation for the relatively high diversity of Bacteroidetes. We consider a cell bank explanation more likely than niche variety as an explanation for the enormous diversity because the crusts are rather homogenous due to the mechanism of salt accumulation.

### Drivers of Community Composition

Our statistical analyses of community composition and abundance patterns showed that not only are samples from a given site most similar to each other, but also that samples from adjacent sites are more similar to each other than samples from non-adjacent sites. Further, we found that the spatial distance between samples correlates with the degree of difference between the microbial communities. The finding that moisture and longitude (proximity to the coast) have the highest correlation with the observed microbial community structure is not surprising, as this is an extremely arid system. Our work supports other reports from hyperarid systems. For example, Robinson et al. (2014) noted that the abundance of algae at sites in the hyperarid region of the Atacama Desert was correlated to fog occurrence, and Pointing et al. (2007) found that species richness and diversity were positively correlated to the availability of liquid water in hypolithic communities in a hyperarid desert in China.

Only at higher moisture levels did we find the most complex communities. At the wettest site, we detected a novel Thermoplasmatales, which has not previously been identified in Atacama salt crusts. Further, the greatest abundance and diversity of Parcubacteria and Nanohaloarchaea occurred at the wettest site. We suggest that the fog level at this site exceeds some threshold water activity, reducing their stress sufficiently and enabling their survival. Our data indicate that dispersal does not control community membership (as seen for Cyanobacteria); rather moisture level, controlled by proximity to the coast, determines community composition (**Figure 5**, **Table 1**, and Supplementary Table 2).

### Atmospherically Derived Carbon and Nitrogen

Cyanobacteria and algae are inferred to be the primary producers for these communities. The decline in the quantity of primary producers along the fog gradient indicates that the wettest site supports the highest capacity for photosynthesis. Radiocarbon measurements of organic carbon in the crusts at the intermediate and driest sites similarly found that carbon cycling occurs much more rapidly at the intermediate site compared to the driest site (Finstad et al., 2016).

We were unable to identify any genes involved in a nitrogen fixation pathway, indicating that nitrogen fixation is not occurring in the salt crusts. A lack of nitrogen fixation has been suggested by others studying lithic communities in extreme environments, however, data limitations have impeded the ability to confirm this hypothesis. For example, Friedmann and Kibler (1980) incubated endolithic communities from a range of hot and cold desert environments and found that the ability to fix nitrogen was the exception. They proposed that because productivity and growth in these environments are very low, abiotic sources of nitrogen are sufficient to sustain the communities. Similarly, Crits-Christoph et al. (2016) did not identify *nif*-genes from halite communities in a nearby salt flat and Pointing et al. (2007) did not find them in datasets from hypolithic communities in a hyperarid region of China. Given the extensive metagenomic dataset examined here and the geographic area surveyed, this strongly suggests that no organisms capable of nitrogen fixation are present in salt crust communities of the hyperarid Atacama Desert. This region has long been known for its large nitrate deposits which are believed to be atmospherically derived (Michalski et al., 2004). We hypothesize that the assimilation of atmospherically deposited nitrate and ammonium are the main source of nitrogen for these communities.

## Conclusion

We found that the Atacama Desert salt crusts host communities that are sustained by cyanobacterial- and algal-based CO_2_ fixation, and suggest that they derive bioavailable nitrogen from external sources as they are not capable of nitrogen fixation. The communities are dominated by a large variety of Halobacteriales and Bacteroidetes species, a few Cyanobacterial populations (Halothece and Euhalothece), and Chlorophyta. These communities harbor an unexpected diversity of organisms at lower abundance based on previous studies, including members of the Candidate Phyla Radiation (Parcubacteria and Saccharibacteria) and Marine Group II Thermoplasmatales, which have never before been reported in hypersaline environments. Further, we detected multiple Nanohaloarchaea and report one complete genome. This group of archaea was only recently first reported from Atacama salt crusts (Crits-Christoph et al., 2016). There is a remarkable contrast between the extremely high sample specificity of most community members and the widespread distribution of a single very closely related Halothece. The genetic similarity of the Halothece among all samples indicates an important role of inter-site dispersal for this key community member. We conclude that selection due to moisture availability is the main driver of community membership and the observed abundance patterns.

## Author Contributions

KF and RA contributed to study design and sampling; KF, AP, BT, GA, CD, AE, and JB contributed to data acquisition and interpretation; KF and JB drafted the manuscript; all authors contributed to editing and revising the manuscript; all authors approved this version for submission.

## Conflict of Interest Statement

The authors declare that the research was conducted in the absence of any commercial or financial relationships that could be construed as a potential conflict of interest.

## Footnotes

1 http://github.com/najoshi/sickle

2 http://bioinformatics.oxfordjournals.org/content/26/19/2460

3 http://ggkbase.berkeley.edu

4 http://www.jcvi.org/cgi-bin/tigrfams/Listing.cgi

5 http://hmmer.org/

6 http://crispr.i2bc.paris-saclay.fr/
